# Identifying key transcription factors and immune infiltration in non-small-cell lung cancer using weighted correlation network and Cox regression analyses

**DOI:** 10.3389/fonc.2023.1112020

**Published:** 2023-05-01

**Authors:** Jingyao Zhang, Yinuo Wang, Baowen Yuan, Hao Qin, Yong Wang, Hefen Yu, Xu Teng, Yunkai Yang, Jun Zou, Min Zhang, Wei Huang, Yan Wang

**Affiliations:** ^1^ Key Laboratory of Cancer and Microbiome, State Key Laboratory of Molecular Oncology, National Cancer Center/National Clinical Research Center for Cancer/Cancer Hospital, Chinese Academy of Medical Sciences and Peking Union Medical College, Beijing, China; ^2^ Department of Ultrasound, National Cancer Center/National Clinical Research Center for Cancer/Cancer Hospital, Chinese Academy of Medical Sciences and Peking Union Medical College, Beijing, China; ^3^ Beijing Key Laboratory of Cancer Invasion and Metastasis Research, Department of Biochemistry and Molecular Biology, School of Basic Medical Sciences, Capital Medical University, Beijing, China

**Keywords:** Transcription Factors, WGCNA, Cox regression analysis, LASSO analysis, Immune infiltration, NSCLC

## Abstract

**Introduction:**

Lung cancer is one of the most common cancers and a significant cause of cancer-related deaths. Non-small cell lung cancer (NSCLC) accounts for about 85% of all lung cancer cases. Therefore, it is crucial to identify effective diagnostic and therapeutic methods. In addition, transcription factors are essential for eukaryotic cells to regulate their gene expression, and aberrant expression transcription factors are an important step in the process of oncogenesis in NSCLC.

**Methods:**

Differentially expressed transcription factors between NSCLC and normal tissues by analyzing mRNA profiling from The Cancer Genome Atlas (TCGA) database program were identified. Weighted correlation network analysis (WGCNA) and line plot of least absolute shrinkage and selection operator (LASSO) were performed to find prognosis-related transcription factors. The cellular functions of transcription factors were performed by 5-ethynyl-2'-deoxyuridine (EdU) assay, wound healing assay, cell invasion assay in lung cancer cells.

**Results:**

We identified 725 differentially expressed transcription factors between NSCLC and normal tissues. Three highly related modules for survival were discovered, and transcription factors highly associated with survival were obtained by using WGCNA. Then line plot of LASSO was applied to screen transcription factors related to prognosis and build a prognostic model. Consequently, *SETDB2, SNAI3, SCML4*, and *ZNF540* were identified as prognosis-related transcription factors and validated in multiple databases. The low expression of these hub genes in NSCLC was associated with poor prognosis. The deletions of both *SETDB2* and *SNAI3* were found to promote proliferation, invasion, and stemness in lung cancer cells. Furthermore, there were significant differences in the proportions of 22 immune cells between the high- and low-score groups.

**Discussion:**

Therefore, our study identified the transcription factors involved in regulating NSCLC, and we constructed a panel for the prediction of prognosis and immune infiltration to inform the clinical application of transcription factor analysis in the prevention and treatment of NSCLC.

## 1 Introduction

Lung cancer is the leading cause of cancer-related morbidity and mortality. It is estimated that there will be nearly 2.4 million new lung and bronchus cancer cases and 1.3 million deaths per year, accounting for nearly 21.4% of cancer-related deaths (1). Non-small cell lung cancer (NSCLC) accounts for approximately 85% of lung cancer cases (2), and it mainly includes lung adenocarcinoma (LUAD) and lung squamous cell carcinoma (LUSC) (3). Surgical treatment is currently the most important means of achieving local disease control and curing stage I–III and oligometastatic NSCLC (4). Low-dose chest computed tomography (CT) in patients with NSCLC has reduced lung cancer mortality by approximately 6.7%. The use of immune checkpoint inhibitors (CPIs) has profoundly promoted the development of novel, effective NSCLC treatments (5). Despite therapeutic advances in surgery, chemotherapy, immunotherapy, and radiation, prognoses of patients with advanced NSCLC remains unsatisfactory (5, 6). Recent studies have shown different driver gene mutations and gene expression characteristics in NSCLC (7). Molecular characterization and utility of immunohistochemistry biomarkers in NSCLC precision medicine are a focus of current research (7). Particularly, RNA-sequencing (RNA-seq) is vital in cancer outlining because of the rapid development of next-generation genome-sequencing technology.

Tumors are cells with unlimited proliferation ability, and they are surrounded by the tumor microenvironment (TME) (8). The TME comprises diverse elements, including cancer cells, immune cells, tumor-associated fibroblasts, and various inflammatory cytokines secreted by these cells, such as interleukin 6 (IL6), vascular endothelial growth factor (VEGF), transforming growth factor β1 (TGF-β), and cytochrome P450 family 1 subfamily B member 1 (CYP1B) (9, 10). Tumor initiation and progression are largely affected by TME dynamics and spatial and temporal heterogeneity (11, 12), and the TME plays a vital role in NSCLC (13). Treatment of NSCLC has been revolutionized by applying programmed cell death 1 (PD-1)- and programmed cell death 1 ligand 1 (PD-L1)-related immune CPIs (14). The PD-1/PD-L1 inhibitors are the standard first-line treatments for metastatic NSCLC (15).

With the continuous development of treatments, immunotherapy resistance is a problem that must be urgently solved (16). A key strategy in counteracting CPI resistance is modulation of immunosuppressive TME. The TME is a mediator of the interaction between tumor and immune cells with multiple important roles, and it affects the response to immunotherapy (17). Resistance to inhibitors of immune examination is suppressed by regulating factors and receptors in the TME. For example, ectopic expression of tumor-associated macrophages (TAM) receptors in NSCLC may contribute to an immunosuppressive, tumor-promoting TME; TAM receptor inhibitors (TAM RI) convert immunosuppressive TMEs into immunostimulatory TMEs to overcome CPI resistance in NSCLC (14). Lefitolimod, an agonist of Toll-like receptor 9 that promotes innate and adaptive immune responses, converts non-immunogenic (“cold”) tumors into immunogenic (“hot”) tumors. Lefitolimod and CPI combination produces synergistic antitumor potency (18). C-C Motif Chemokine Receptor 4 (CCR4) inhibitors, which mediate tumor trafficking of regulatory T cells (Tregs), lead to decreased Treg frequency and increased antitumor activity; however, CCR4 inhibitors are used in combination with CPI to improve antitumor efficacy (16). Consequently, exploring the regulatory mechanism of TME is crucial for clarifying the mechanism of tumorigenesis and addressing the problems of immunotherapy sensitivity and drug resistance.

Transcription initiation in eukaryotic cells is a complex process involving binding transcription factors (TFs) (19). Transcription factors have been implicated in each stage of the development and progression of various human tumors. Many studies have shown that TFs play critical roles in cancer; for example, they influence the activity of genes involved in immunological response, immune cell infiltration, and the cell cycle (19, 20). Moreover, TFs can be products of oncogenes or tumor suppressor genes. Epithelial-mesenchymal transition (EMT)-activating TFs promote tumorigenesis and cancer invasion in cell lines and xenograft mice models (21). Recent studies have shown that some TFs predict the prognosis of patients with malignant tumors. Other studies have shown that forkhead box TFs affect the EMT and regulate hormones and the immune system, affecting tumor development, metastasis, and drug resistance (22). Manshouri et al. reported that ZEB1, the TF of the EMT, recruits the nucleosome remodeling and deacetylase complex in NSCLC, acts as a transcriptional suppressor, and transcriptionally inhibits *TBC1D2b* expression, promoting the E-cadherin degradation and mesenchymal tumor transition (23). Mollaoglu et al. reported that tumor-associated neutrophils in NSCLC recruit SOX2 to mediate *CXCL5* expression and regulate the TME (24). While TFs have attracted much attention in tumor research, their roles in tumor prognosis remain unclear.

Weighted correlation network analysis (WGCNA) is used to identify clusters (modules) of highly related genes. It can be used to evaluate gene interconnectivity in the module and convert gene expression profile data into co-expression modules, allowing for the construction of a gene regulatory network. There are five steps in WGCNA: constructing a gene co-expression network, identifying modules, relating modules to external information, studying module relationships, and finding the key drivers of interesting modules (25). This method is widely used in research of many cancers, including pancreatic (26), lung (27), and liver cancers (28). However, differential expression analysis only considers each gene’s expression, whereas gene network analysis considers the connections between genes, building a more comprehensive network of tumor regulatory mechanisms (29).

In the present study, a prognostic model of NSCLC based on cancer-related TFs and the effect of TFs on NSCLC prognosis was constructed. The mRNA expression of NSCLC using The Cancer Genome Atlas (TCGA) dataset was analyzed. Based on the TCGA-NSCLC gene expression profile, differential genes were obtained through differential expression analysis between cancer and normal samples. A panel of five survival-associated TFs was identified. This study will be significant in the exploration of TFs related to the prognosis of potential NSCLC and the role of TFs in NSCLC.

## 2 Materials and methods

### 2.1 Antibodies and reagents

Antibodies used in this study included Vimentin (#5741), SLUG (#9585), β-catenin (#8480), and Anti-rabbit Alexa Fluor^®^ 594 Conjugate (#8889) from Cell Signaling Technology and MMP9 (10375-2-AP), SNAI1 (13099-1-AP), SNAI3 (21350-1-AP), and SETDB2 (14428-1-AP) from Proteintech. The siRNAs were obtained from Shanghai GenePharma.

### 2.2 Cell culture and transfection

The cell lines were purchased from American Type Culture Collection (ATCC). The cell lines BEAS-2B, A549, HCC827, H1299, H1975, EPLC-272H, H226, H157, and H2170 were cultured in a humidified incubator at 5% CO_2_ and 37°C using RPMI1640 medium with 10% fetal bovine serum (FBS) and 1% Pen-Strep penicillin-streptomycin (Gibco). Transfections were performed with RNAiMAX Reagent (Invitrogen) according to the manufacturer’s instructions. Each experiment was performed at least three times. siRNA against SETDB2 (5’-GUGUACGCUGUCUAGAUGATT-3’), siRNA against SNAI3 (5’-GACGCAGAGAGAAAUCAAUTT-3’) and siRNA negative control (5’-UUCUCCGAACGUGUCACGUTT-3’) were obtained from Shanghai GenePharma.

### 2.3 Database

**Figure 1:**
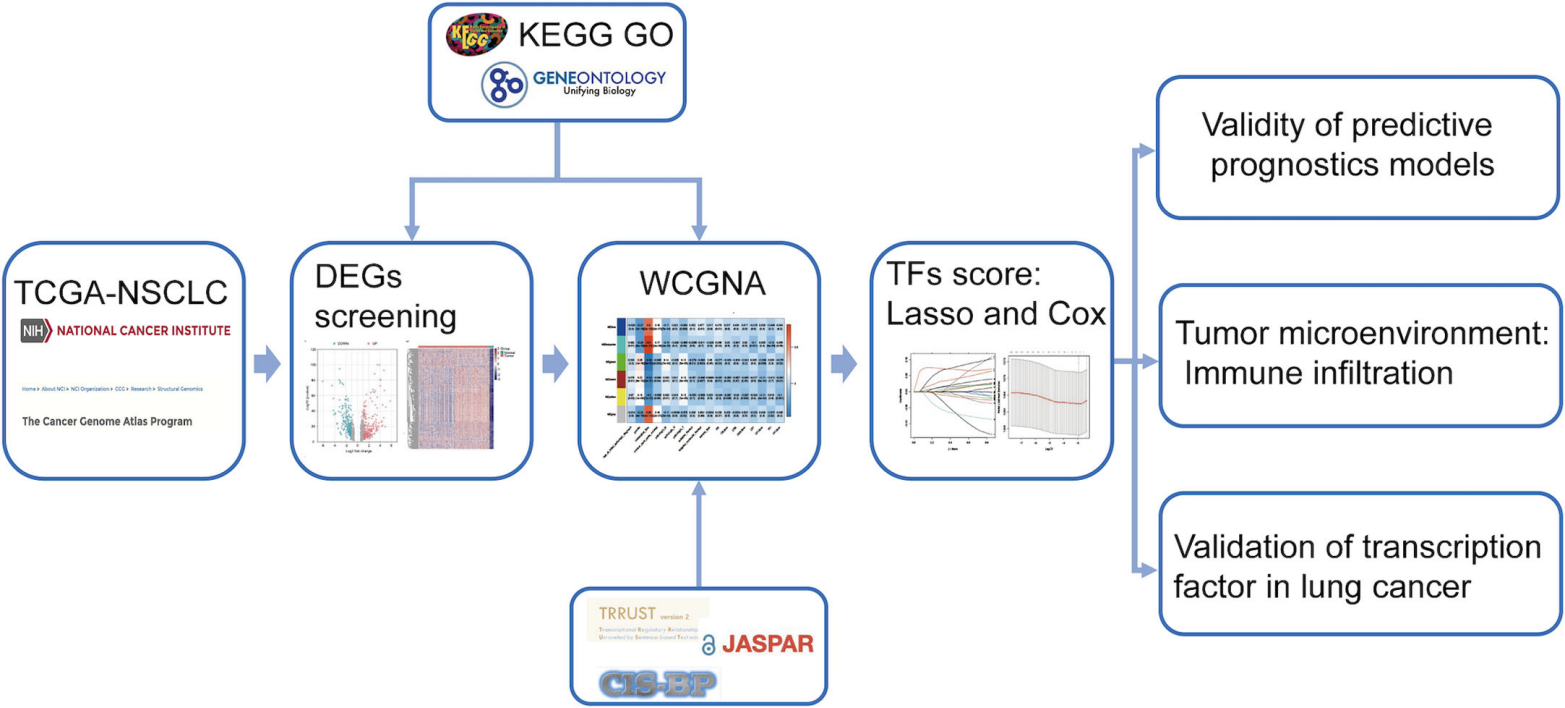
Workflow of data analysis.

The research route is shown in **Figure 1**. The TCGA dataset referenced in the study is available in a public repository from UCSC Xena (https://xena.ucsc.edu/), including transcriptome expression RNA-seq and clinical data of 1076 NSCLCs. The expression profile and survival information of patients with NSCLC, including 130 samples, were downloaded from the UCSC database (https://ucscpublic.xenahubs.net). Transcription factor sets were defined using Trrust (https://www.grnpedia.org/trrust/), CISBP (http://cisbp.ccbr.utoronto.ca/index.php), and JASPAR (http://jaspar.genereg.net/). In total, 1942 (after de-redundancy) human TFs were downloaded from the database for subsequent analysis.

Data from 130 samples from UCSC were used to verify the efficacy of the TFs prognostic scores. After standardizing the maximum and minimum data values, a log-rank test was performed on the overall survival (OS) data, and a Kaplan–Meier curve was drawn. *p* < 0.05 was considered a statistically significant correlation. The survival receiver operating characteristic (ROC) software package was used to plot the ROC curve, observe the prognostic efficacy with 5-year survival as the threshold, and calculate the area under the curve (AUC) score. The ROC curve was used to describe the sensitivity and specificity of the survival prediction based on the TFs score.

### 2.4 Differential gene expression

Differentially expressed genes (DEGs) between normal and tumor samples were estimated using the limma R package. Using a Bayesian approach to computation, candidate DEGs were identified by *p <* 0.05 and |fold-change| > 1.5. After determining the intersection with the TF set, the “pheatmap” R package was used to draw a heatmap of partial differential TF gene expression in tumor and normal tissues. Gene Ontology (GO) and Kyoto Encyclopedia of Genes and Genomes (KEGG) enrichment analyses were performed using the Database for Annotation, Visualization, and Integrated Discovery (DAVID: https://david.ncifcrf.gov/).

### 2.5 Co-expression network construction

The “WGCNA” R package (25) was used to perform co-expression network analysis on TCGA containing differentially expressed TFs. First, no abnormal samples were found during sample clustering. A picksoft threshold was used to select the optimal soft threshold β for subsequent network construction; the expression profile was analyzed using scale-free clustering and a dynamic shearing tree to identify co-expressed gene modules. The co-expression network matrix calculation was used on the Pearson correlation. In addition, the relationship between these gene modules and clinical phenotypes was positioned the module as a survival module; the genes of these modules were used as candidate prognostic-related TFs.

### 2.6 Cox regression and line plot of least absolute shrinkage and selection operator regression

For the cancer samples of TCGA-NSCLC (997 samples after removing the no-OS data), the TF genes were analyzed using Cox single-factor survival analysis based on the survival package; the prognostic genes with single-factor significance (*p* < 0.05) were selected as candidate genes. The Kaplan–Meier method was used to establish survival curves, and the log-rank test was used to calculate the significance of the differences. Forest plot software was to plot a prognostic forest plot of the Cox single-factor results of related genes. Subsequently, prognosis-related TFs were included in the LASSO regression analysis using the “glmnet” R package to obtain the prognostic-related TFs, obtain the corresponding genes’ regression factor parameters, and build a TFs prognosis model based on this. Finally, Cox proportional hazards regression was used to analyze the independent predictive power of the TFs score.

### 2.7 Quantitative reverse transcription PCR

Total cellular RNA was extracted from cells using an RNA-Quick Purification Kit (Yishan Biotechnology). cDNA was prepared using a Transcriptor First Strand cDNA Synthesis Kit (Roche, Basel, Switzerland). Reverse transcriptase polymerase chain reaction of the selected genes was performed by reverse transcriptase polymerase chain reaction using ABI QuantStudio 5 (Applied Biosystems). In addition, SYBR Green fluorescence was measured and quantified using the comparative Ct method (2^-^ΔΔCt), with *GAPDH* expression as an internal control. The assay was performed in triplicate, and the primers used are listed in **Table 1**.

**Table 1:** The primers of select genes.

Gene	Forward primer	Reverse primer
*ETV1*	GGCCCCAGGCAGTTTTATGAT	GATCCTCGCCGTTGGTATGT
*SCML4*	TCACTCCACGCCTATGAAGAT	GGGTTTCCGCCCTCTTTTC
*SETDB2*	ACCACCCCGAGAGCATCTGAACT	TGTGGTCGCCTGGTTACATCTGC
*SNAI3*	ACTGCGACAAGGAGTACACC	GAGTGCGTTTGCAGATGGG
*ZNF540*	AGTGTATGCGGACAACTTACCC	AGGTTTCTTACCTGCATGAGTTC
*MMP9*	CGCAGACATCGTCATCCAGT	AACCGAGTTGGAACCACGAC
*Vimentin*	AGGCAAAGCAGGAGTCCACTGA	ATCTGGCGTTCCAGGGACTCAT
*β-catenin*	AAAGCGGCTGTTAGTCACTGG	CGAGTCATTGCATACTGTCCAT
*SNAI1*	TGCCCTCAAGATGCACATCCGA	GGGACAGGAGAAGGGCTTCTC
*SLUG*	ATCTGCGGCAAGGCGTTTTCCA	GAGCCCTCAGATTTGACCTGTC
*SOX2*	GCCTGGGCGCCGAGTGGA	GGGCGAGCCGTTCATGTAGGTCTG
*OCT4*	ATCACCCTGGGATATACACAG	CTGCTTTGCATATCTCCTGA
*NANOG*	TCTGGACACTGGCTGAATCCT	CGCTGATTAGGCTCCAACCAT
*GAPDH*	GTCAACGGATTTGGTCGTAT	GTCAACGGATTTGGTCGTAT

### 2.8 EdU assay

Cells were inoculated into 12-well plates. Next, the cells were processed using the EdU cell proliferation kit (C10310 RiboBio). Finally, the assay was performed with a fluorescent microscope. The whole process was carried out according to the manufacturer’s instructions.

### 2.9 Wound healing assay

Cells were taken at logarithmic growth stage, spread in a 6-well plate, and waited for the cells to grow above 95% confluence for scratching. Cells were washed 3 times with PBS and photographed; at this point the photo was taken for 0 h. The cells were added to the medium and incubated, and the healing of the scratches was observed and photographed. The images were used to analyze the percentage of healing of the scratches. Images at time zero (0 h) and at 24 or 36 h (Δ h) were captured. The area of wound was quantified by Image J software. The percentage of wound closure: percentage of wound closure = [(A (0 h) – A (Δ h)/A (0 h)] × 100%, A (0 h) is the area of wound measured immediately after scratching, and A (Δ h) is the area of wound measured 24 or 36 h after scratching.

### 2.10 Cell invasion assay

Transwell chamber filters were wrapped with Matrigel (BD Biosciences, Franklin Lakes, NJ). Lung cancer cells transfected with specific siRNAs were suspended in serum-free medium, and the cells were seeded into the upper chamber of the Transwell. The lower chamber was filled with medium containing 10% FBS. After 24 h of incubation, the cells were fixed with methanol and stained with crystal violet solution, after which the cells in the upper chamber were wiped off with a cotton swab and the remaining cells were photographed. Five high-power fields of view were taken for each small chamber selection.

### 2.11 Immunofluorescence staining

Cells were cultured in confocal-specific dishes until 85% confluence was reached, when they were fixed in 2% paraformaldehyde fixative, after which the cells were washed 3 times with PBS. Cells were treated with 0.2% triton x-100 for 5 min at 25°C, washed 3 times with PBS, and closed with 0.8% bovine serum albumin for 1 h at 25°C. Cells were incubated overnight or for 1 h at 25°C by primary antibody and then they were washed 2 times (10 min each) at 25°C with 0.1% triton x-100. Cells were incubated for 1 h with the addition of secondary antibody, and 0.1% triton x-100 was added to wash twice for 10 min each time at 25°C. Finally, fluoroshield with 4′,6-diamidino-2-phenylindole (DAPI) was added to cover the bottom of the dish. The mean fluorescence intensity (MFI) of cells was calculated from Image J software.

### 2.12 Western blotting

Cell-culture dishes were placed on ice, and cells were washed with ice-cold PBS. Cells were added to ice-cold Radio Immunoprecipitation Assay (RIPA) lysate and lysed at 4°C for 10 min, vortexing every 5 min. The BCA was quantified, and protein lysate was added to the loading buffer for 10 minutes at 95°C. Electrophoresis was carried out at 80 V until bromophenol blue ran through the top layer of the gel, followed by a shift to 120 V until bromophenol blue ran out of the sodium dodecyl sulfate polyacrylamide gel electrophoresis (SDS-PAGE) gel. To transfer the proteins from the gel to the membrane, the polyvinylidene fluoride (PVDF) was activated with methanol for 1 min and rinsed with transfer buffer in a “sandwich” filter paper and sponge configuration, sponge–filter paper–gel-membrane filter paper–sponge. The PVDF membrane was placed in 5% skimmed milk for 1 h at 25°C. The PVDF membranes were washed with tris buffered saline with tween 20 (TBST), and then primary antibody (diluted in primary antibody diluent) was added and incubated overnight at 4°C. The membranes were washed 3 times in TBST for 5 minutes each time, secondary antibody prepared in 5% skimmed milk was added, and the membranes were incubated for 1 h at 25°C in a gentle mix. Membranes were washed 3 times in TBST for 5 minutes each time. Chemiluminescence images were obtained using a darkroom development technique. Western Blot signal was quantified using the Image J software.

### 2.13 Statistical analysis

GraphPad Prism (version 8.0) was used to analyze the data from our study. Results are expressed as the mean ± SD of at least three individuals. Student’s t-test was used to compare differences between the two groups. Survival curves were plotted using the Kaplan–Meier “survival” package in R (version 3.4.3). Log-rank test was used to assess statistical significance. Statistical significance was set at *p* < 0.05.

## 3 Results

### 3.1 Identifying differentially expressed TFs in NSCLCs and normal samples

**Figure 2:**
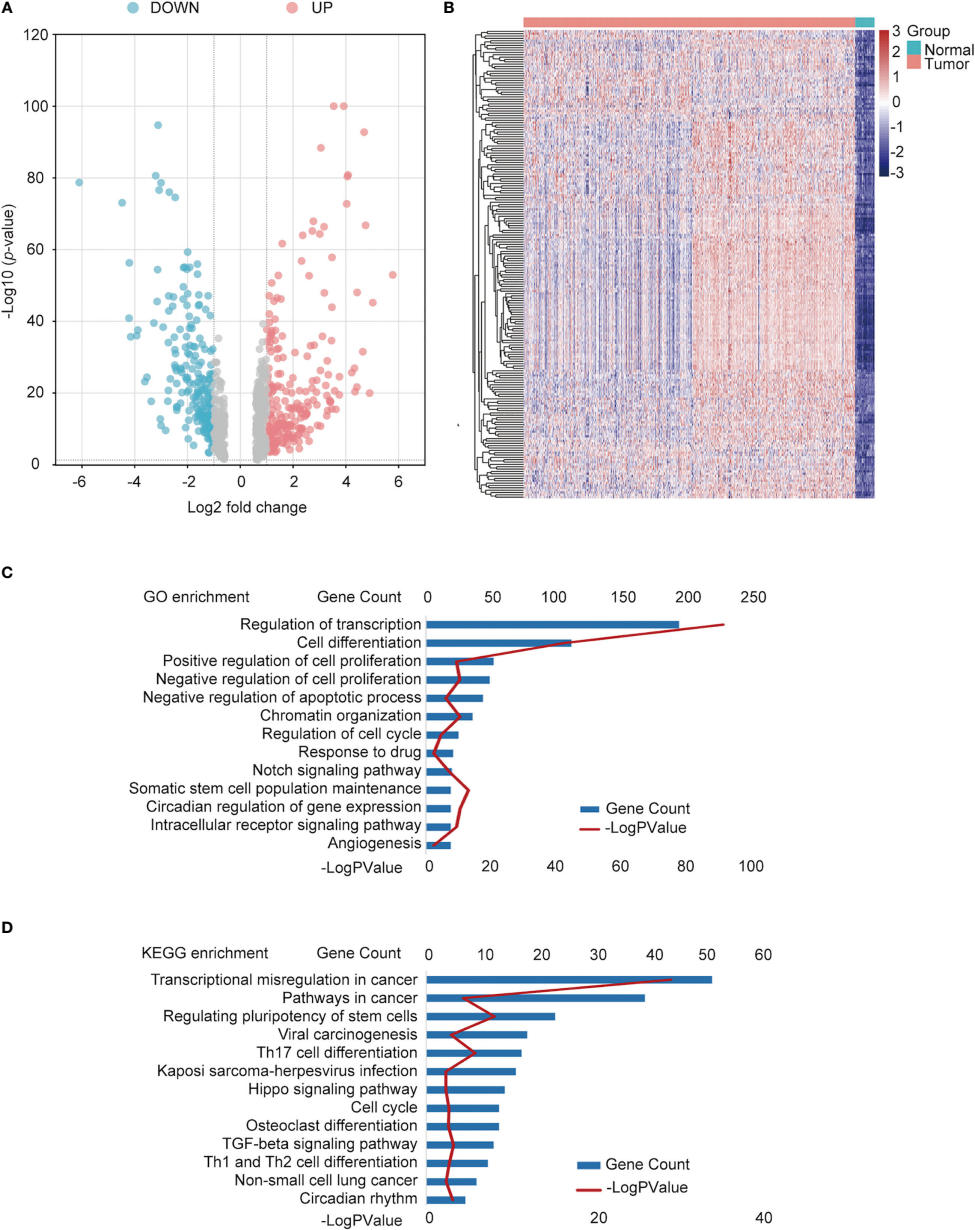
Identification of differentially expressed TFs in NSCLC and normal tissues. **(A)** Volcano plot of differentially expressed TFs. Red points: upregulated genes; Blue points: downregulated genes. **(B)** Heatmap of the top upregulated TFs. Green square group: normal tissues; pink square: tumor tissue. **(C)** Functional annotation of the TFs using Gene Ontology (GO) term. **(D)** Kyoto Encyclopedia of Genes and Genomes (KEGG) pathway enrichment of differential TFs.

Data containing 1017 NSCLC and 59 normal samples from the TCGA dataset with clinical and RNA-seq datasets were used. Differential analysis was performed to find differentially expressed genes (DEGs) between normal and NSCLC samples, with the criteria of |fold change| >1.5 and *p* < 0.05; 8840 DEGs were identified. In total, 725 TFs (**Supplemental Table 1**) were identified as NSCLC-related TFs (**Figure 2A**), of which the top 200 TFs were upregulated in NSCLC compared to normal tissues. The heatmap of the top 200 differentially expressed TFs is shown in **Figure 2B**. To further determine the function of these TFs, GO and KEGG pathway annotations were used to analyze the 725 differential TFs. These genes could be enriched in tumor malignancy-related GO biological processes, containing cell differentiation, proliferation, apoptosis, stemness, and angiogenesis (**Figure 2C**). Moreover, KEGG pathway enrichment analysis showed that 725 genes were involved in many pathways, including tumor transcriptional regulation interrelated to oncogenesis and development, the Hippo signaling pathway, TGF-β signaling pathway, cell cycle, immune-related Th17 cell differentiation, and Th1 and Th2 cell differentiation (**Figure 2D**).

### 3.2 Construction of NSCLC co-expression module *via* WGCNA

**Figure 3:**
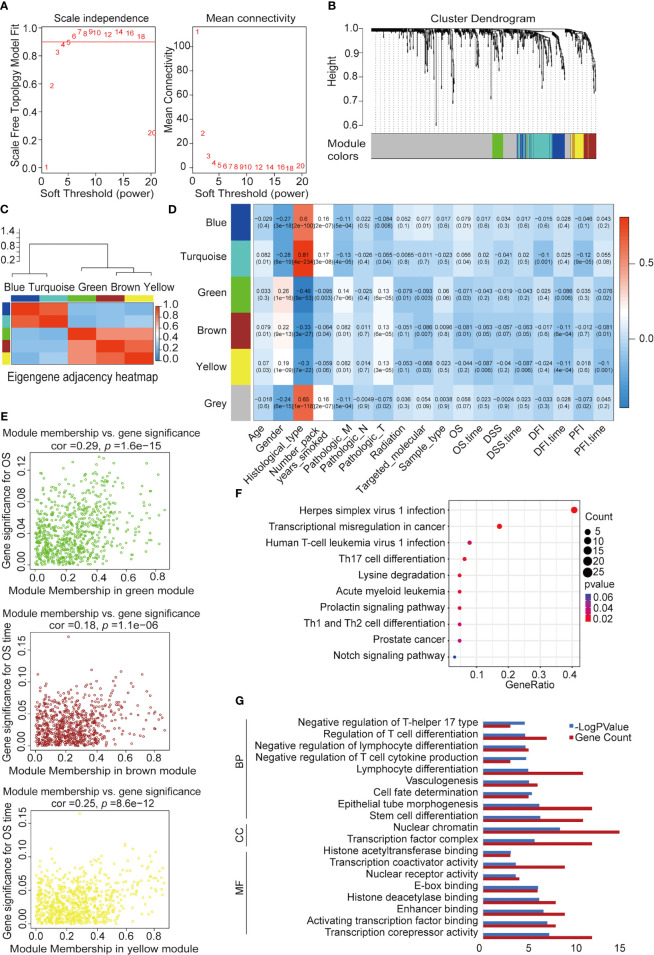
Construction of NSCLC co-expression module *via* WGCNA. **(A)** Analysis of network topology for various soft-threshold powers. Left: The relationship between the soft threshold β and the scale-free network topology R^2^, where the threshold is set to 0.9 for R^2^ and the β value standard is the corresponding β value when R^2^ reaches 0.9 for the first time. Right: Relationship between the soft threshold β and connectivity. **(B)** Clustering dendrograms of TFs with dissimilarity based on topological overlap with assigned module colors. **(C)** Heatmap visualization of correlations between modules. **(D)** Analysis of module-NSCLC clinical data relationships. **(E)** Co-expression analysis of gene, brown, and yellow significance (GS) and module membership (MM). **(F)** Kyoto Encyclopedia of Genes and Genomes (KEGG) pathway enrichment of 118 TFs. **(G)** Functional annotation of 118 TFs using Gene Ontology (GO) term. MF, molecular function; CC, cellular component; BP: biological process.

We used WGCNA to build a co-expression network and modules of NSCLC-correlated differentially expressed TFs. A co-expression network was constructed with several modules. A scale-free network evaluation coefficient threshold > 0.9 was selected to make the co-expression network conform to the scale-free network standard. The 725 transcription factors were subjected to clustering analysis to classify them into six modules (**Figures 3A, B**) (**Table 2**). The six modules were divided into two categories: green, brown, and yellow made up one category, while the other category included blue and turquoise (**Supplemental Table 2**). Internal modules in the same category were positively correlated (**Figure 3C**). The correlation analysis of each module and clinical data was performed to find transcription factor modules that are highly correlated with clinical survival. There was a correlation between the gene significance of OS and module membership of the green module (cor = −0.071, *p* = 0.03). The OS time negatively correlated with “brown” (cor = −0.063, *p* = 0.05) and “yellow” (cor = −0.087, *p* = 0.006) (**Figure 3D**). The module membership of the green module was significantly related to the gene significance of the OS, and the module membership of the brown and yellow modules was significantly related to the gene significance of the OS time (**Figure 3E**). Therefore, 118 genes contained in these three modules (green, brown, and yellow) were selected as candidate survival-related TFs for further analysis. 118 genes were enriched in many immune-related pathways, including T-cell differentiation, lymphocyte differentiation, and T-cell cytokine production. Furthermore, the potential regulatory molecular functions of these TFs were found to involve their classical functions, including enhancer binding, histone deacetylation, nuclear receptor activity, and histone acetyltransferase (**Figures 3F, G**) (**Supplemental Table 3**).

**Table 2:** The number of genes in the coexpression modules.

Module	Gene count
Blue	64
Brown	43
Green	36
Turquoise	85
Yellow	39
Grey	458

### 3.3 Construction of Prognostic TFs in NSCLC

**Figure 4:**
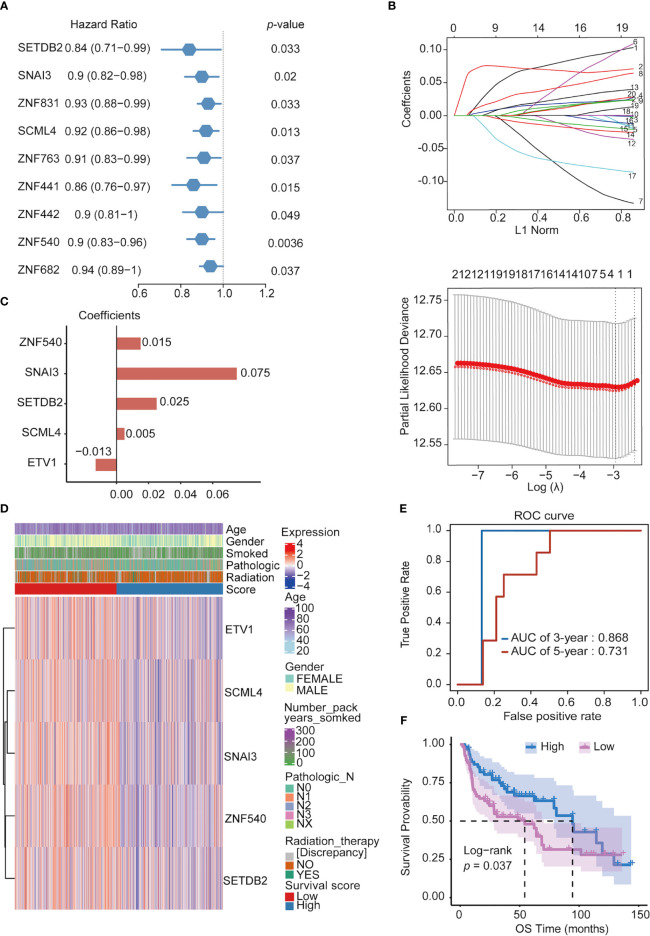
Constructing prognostic TFs in NSCLC. **(A)** Univariate Cox regression analysis of TFs. **(B)** Line plot of least absolute shrinkage and selection operator (LASSO) regression analysis of 30 TFs in NSCLC. **(C)** Coefficients of relevant 5 TFs in LASSO regression result. **(D)** Heatmap of the five prognosis-related TFs expression profiles combined with clinical traits in the high- and low-score groups. **(E)** ROC curve of 3- and 5- year survival probability in NSCLC cancer from the UCSC dataset. **(F)** Kaplan–Meier analysis of high- and low-score groups for NSCLC from the UCSC dataset.

To search for TFs with key roles in the NSCLC prognosis, Cox single-factor regression analysis was performed on the above 118 key TFs; 9 TFs significantly (*p* < 0.05) related to survival were selected (**Figure 4A**). According to previous research, 21 genes highly related to NSCLC cell proliferation and OS of patients with NSCLC in the study were added, including 11 potential tumor suppressor genes (*AFF3, AhR, AR, CBFA2T3, CHD4, KANK2, NR3C2, PTEN, PRDM16, RB1*, and *STK11*) and 10 potential oncogenes (*BARX1, DLX6, ELF3, EN1, ETV1, FOXBE1, IRX4, IRX5*, and *SALL1*) (30). We performed LASSO regression analysis for these 30 TFs. Finally, five prognostic-related TFs (*SETDB2, SNAI3, SCML4, ZNF540*, and *ETV1*) were screened using LASSO regression (**Figure 4B**). The LASSO regression coefficients of each gene were shown in **Figure 4C**. Subsequently, clinical data were analyzed in relation to TFs scores, and the analysis revealed that the number of packs smoked per year (*p* = 0.003017), gender (*p* = 2.899365e-08), pathological stage TNM (T: *p* = 0.0004997501, N: *p* = 0. 006496752, M: *p* = 0.004497751), and targeted molecular therapy (*p* = 0.02204301) had significant differences in the distribution of clinical characteristics in the high and low TFs score groups. Conversely, there was no significant trend in other clinical characteristics (**Figure 4D**).

Furthermore, in the ROC analysis score for prognosis, the 3-year survival AUC value was 0.868, and that for the 5-year survival was 0.731 in the ROC curve (**Figure 4E**). To verify the prognostic model’s validity, the prognostic performance of the TFs score on another set of UCSC (https://ucscpublic.xenahubs.net) data containing 130 samples from NSCLC was analyzed. The TFs score was shown to have a predictive function for the survival of NSCLC patients (*p <* 0.037) (**Figure 4F**).

**Figure 5:**
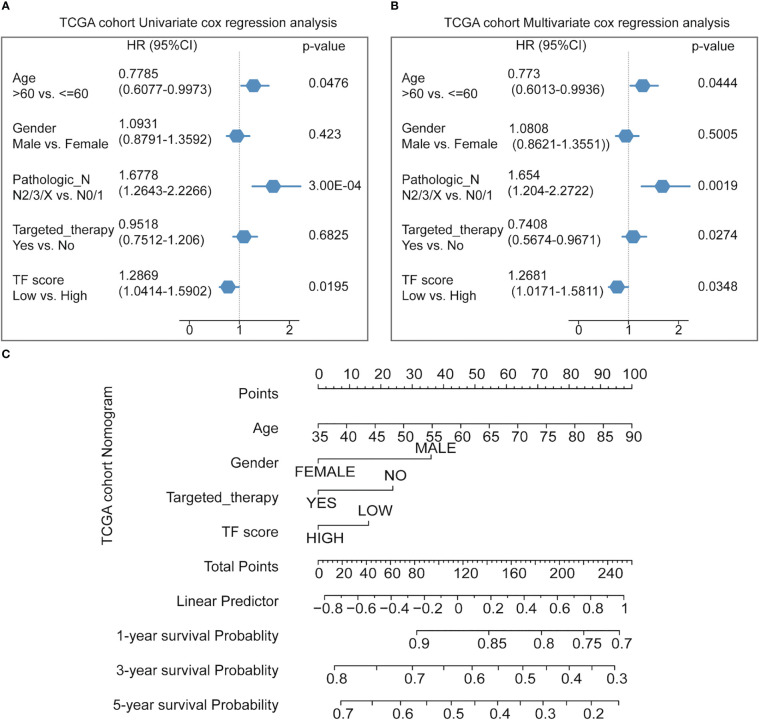
Predicting the prognosis probability in TCGA-NSCLC. **(A)** Univariate Cox regression analysis in the NSCLC cohort. **(B)** Multivariate Cox regression analysis in the NSCLC cohort. **(C)** Nomogram line graph for multivariate cox results of the NSCLC cohort.

Finally, Cox multifactor regression was used to analyze the independent predictive performance of the model and explore the effect of other clinical factors on the prognosis in the TF score. We evaluated the prognostic value of the five TFs according to score, age, sex, number of packs smoked per year, N stage, radiotherapy, and targeted therapy. Univariate regression analysis showed that the TFs score was significantly associated with OS (HR = 1.2869, *p* = 0.0195); multivariate regression analysis showed that the TFs score was significantly correlated with OS (HR = 1.2681, *p* = 0.0348) (**Figures 5A, B**). The nomogram quantified the contribution of individual factors to clinical prognosis to verify the model’s validity, which had good predictive power (**Figure 5C**) (**Supplemental Table 4**).

### 3.4 Analysis of immune infiltration

**Figure 6:**
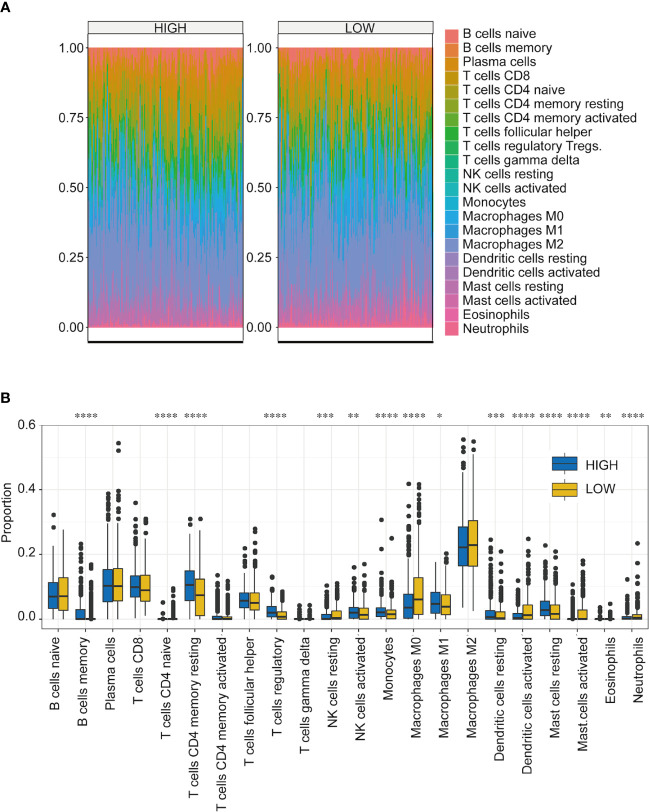
Immune microenvironment analysis of NSCLC in high- and low-score groups. **(A)** Proportion of infiltrating immune cells among the 22 groups with high- and low-TF scores. Different colors represent the cell types. **(B)** Infiltration ratios of different immune cell types with high- and low-TFs score. *p < 0.05, **p < 0.01, ***p < 0.001, ****p < 0.0001. Student’s t-test.

One important function of the immune system is to recognize and subsequently destroy tumors. In recent years, progress in tumor treatment has benefited from research involving immunotherapy (31–33). Therefore, TME plays a vital role in diagnosing and treating tumors. According to the TCGA-NSCLC dataset, we analyzed the differences in the immune microenvironment between the high- and low-score groups (**Figure 6A**). Using CIBERSORT calculations, significant differences were found in the infiltration levels of 22 immune cells in the high- and low-score groups. The immune cells with significant differences were memory B cells, naive CD4 T cells, resting memory CD4 T cells, regulatory T cells, resting natural killer (NK) cells, activated NK cells, monocytes, M0 macrophages, M1 macrophages, resting dendritic cells, activated dendritic cells, resting mast cells, activated mast cells, eosinophils, and neutrophils (**Figure 6B**) (**Supplemental Table 5**).

### 3.5 Gene network of TFs construction and survival analysis of five TFs

**Figure 7:**
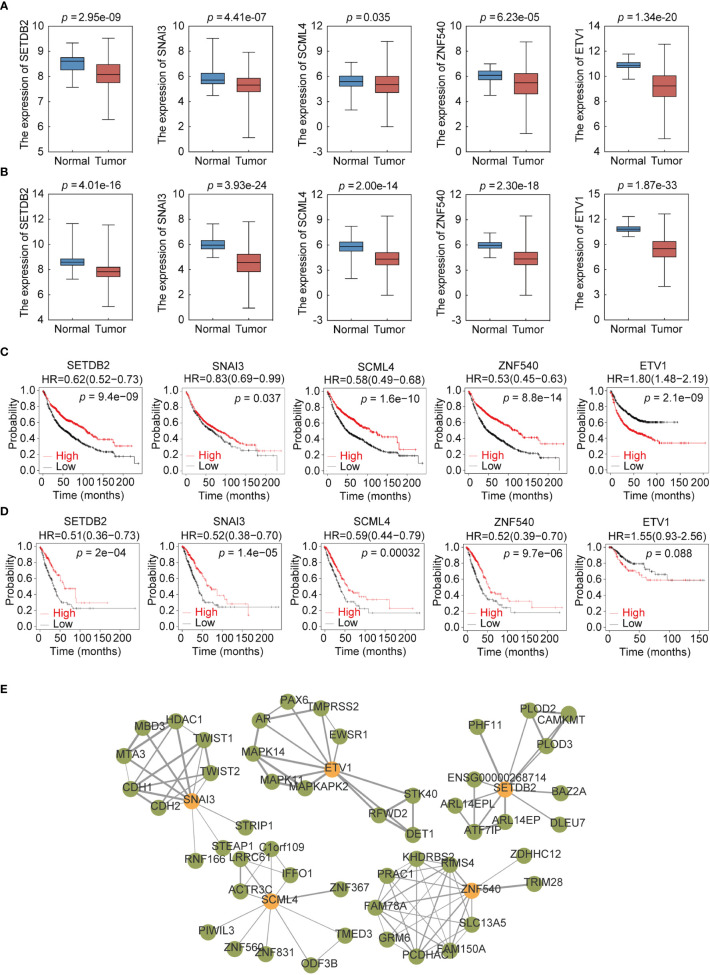
Expression profile and survival analysis of 5 transcription factors (TFs) in NSCLC. **(A)** Expression profile of 5 TFs (SETDB2, SNAI3, SCML4, ZNF540, and ETV1) in LUAD. **(B)** Expression profile of 5 TFs (SETDB2, SNAI3, SCML4, ZNF540, and ETV1) in LUSC. **(C)** Kaplan–Meier analysis of 5 TFs (SETDB2, SNAI3, SCML4, ZNF540, and ETV1) using Affymetrix microarray data. **(D)** Kaplan–Meier analysis of 5 TFs (SETDB2, SNAI3, SCML4, ZNF540, and ETV1) using RNA-seq data. **(E)** Analysis of the 5-TFs protein interaction network.

To further explore the expression of the five TFs in clinical samples, LUAD and LUSC data from TCGA were used to compare their expression in normal and NSCLC samples. The results showed that the expression levels of *SETDB2, SNAI3, SCML4, ZNF540*, and *ETV1* in LUAD (**Figure 7A**) and LUSC (**Figure 7B**) were significantly lower than those in normal tissues. Meanwhile, survival analysis of each TF showed that lower *SETDB2, SNAI3, SCML4*, and *ZNF540* expression was associated with poor prognosis in lung cancer; higher expression of *ETV1* was associated with poor prognosis in lung cancer *via* Affymetrix microarray data and *via* RNA-seq data (**Figures 7C, D**) (https://kmplot.com/analysis). It was worth noting that the expression level of *ETV1* in normal tissues was higher than that in lung cancer tissues; however, patients with high expression had poor prognoses. This phenomenon suggested two roles of ETV1 in the occurrence and development of lung cancer. The TFs likely interacted with other proteins to form complexes that activated or inhibited the transcriptional regulation of genes and downstream genes to function (19). Therefore, the interaction protein network of the five TFs were analyzed using STRING (**Figure 7E**) (**Supplemental Table 6**) (https://string-db.org). These data showed that SETDB2, SNAI3, SCML4, and ZNF540 have tumor suppressor functions in lung cancer.

### 3.6 Validating the TFs phenotypes, functions, and expression in lung cancer

**Figure 8:**
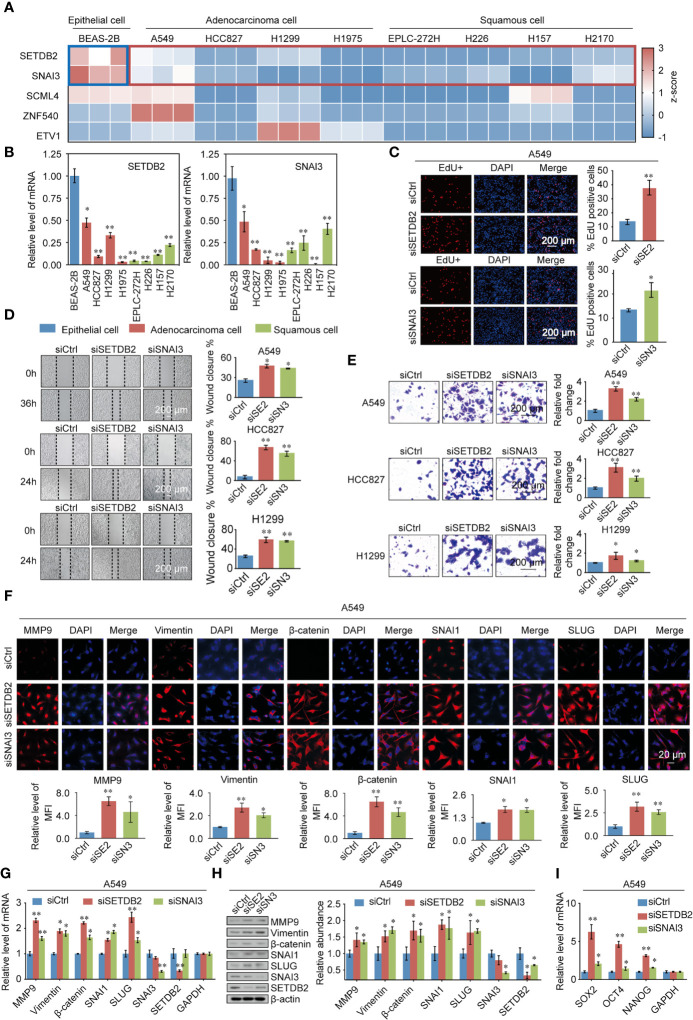
Validating the TFs phenotypes, functions, and expression in lung cancer. **(A)** SETDB2, SNAI3, SCML4, ZNF540, and ETV1 mRNA expression levels in the normal human bronchial epithelial cell line (BEAS-2B), lung adenocarcinoma cell lines (A549, HCC827, H1299, and H1975), and lung squamous cell lines (EPLC-272H, H226, H157, and H2170) using RT-qPCR. Blue box: normal human bronchial epithelial cell line (BEAS-2B). Red box: lung cancer cell lines. **(B)** The mRNA expression levels of SETDB2 and SNAI3 in the normal human bronchial epithelial cell line (BEAS-2B), lung adenocarcinoma cell lines (A549, HCC827, H1299, and H1975), and lung squamous cell lines (EPLC-272H, H226, H157, and H2170) using RT-qPCR. **(C)** EdU assays were performed in A549 cells infected with siCtrl, siSETDB2, and siSNAI3. siCtrl, siControl; siSE2, siSETDB2; siSN3, siSNAI3. **(D)** Wound healing experiments were performed in A549, HCC827, and H1299 transfected with siCtrl, siSETDB2, and siSNAI3. siCtrl, siControl; siSE2, siSETDB2; siSN3, siSNAI3. **(E)** Transwell invasion assays were performed on lung cancer cells (A549, HCC827, and H1299) infected with siCtrl, siSETDB2, and siSNAI3. siCtrl, siControl; siSE2, siSETDB2; siSN3, siSNAI3. **(F)** Immunofluorescence staining including MMP9, Vimentin, β-catenin, SNAI1 and SLUG was performed in A549 cells transfected with siCtrl, siSETDB2 and siSNAI3. Blue fluorescence of DAPI labeled cell nucleus, red fluorescence of labeled corresponding protein. siCtrl, siControl; siSE2, siSETDB2; siSN3, siSNAI3. MFI, mean fluorescence intensity. **(G)** The mRNA expression levels of metastatic markers (MMP9, Vimentin, β-catenin, SNAI1, and SLUG) as well as SNAI3 and SETDB2 were detected by RT-qPCR in A549 cells transfected with siCtrl, siSETDB2, and siSNAI3. siCtrl, siControl. **(H)** The protein levels of metastatic markers (MMP9, Vimentin, β-catenin, SNAI1, and SLUG) as well as SNAI3 and SETDB2 were detected by western blotting in A549 cells transfected with siCtrl, siSETDB2, and siSNAI3. siCtrl, siControl; siSE2, siSETDB2; siSN3, siSNAI3. **(I)** The mRNA expression levels of stem cell markers (SOX2, OCT4, and NANOG) in A549 cells transfected with siCtrl, siSETDB2, and siSNAI3 using RT-qPCR. siCtrl, siControl. **(B-E, G, I)** Error bar represents mean ± SD of three independent experiments. *p < 0.05, **p < 0.01. Student’s t-test.

To gain further support for the notion that SETDB2, SNAI3, SCML4, ZNF540, and ETV1 regulate the malignant phenotypes of lung cancer through the transcriptional repression of genes, experiments were performed. First, to further explore the function of SETDB2, SNAI3, SCML4, ZNF540, and ETV1 in lung cancer cell lines, the mRNA expression levels in the normal human bronchial epithelial cell line (BEAS-2B), lung adenocarcinoma cell lines (A549, HCC827, H1299, and H1975), and lung squamous cell lines (EPLC-272H, H226, H157, and H2170) were measured (**Figure 8A**). Consistent with the results of bioinformatics analysis, the expression levels of SETDB2 and SNAI3 were higher in BEAS-2B than in lung cancer cell lines (**Figure 8B**).

To determine how SETDB2 and SNAI3 regulate the cell proliferation of NSCLC, EdU assays were performed, which showed that knockdown of SETDB2 or SNAI3 in A549 cells had a strong promotion effect on the proliferation of lung cancer cells (**Figure 8C**). We then investigated the roles of SETDB2 and SNAI3 in cellular behavior of NSCLC using wound healing and transwell invasion assay in A549, HCC827, and H1299, which were transfected with control siRNA, SETDB2 siRNA, or SNAI3 siRNA. The wound healing assay showed that depletion of SETDB2 or SNAI3 promoted the migration of lung cancer cells (**Figure 8D**). Meanwhile, the increased invasion was induced by knocking-down SETDB2 or SNAI3 (**Figure 8E**).

We next investigated the possibility of SETDB2 and SNAI3 in NSCLC metastasis and stemness *in vitro*. The result of immunofluorescence assay indicated that metastasis-related markers (MMP9, Vimentin, β-catenin, SNAI1, and SLUG) were significantly upregulated with SETDB2 or SNAI3 knockdown in A549 cells (**Figure 8F**). The expression of metastasis-related markers was analyzed by RT-qPCR and Western blotting using A549 cells transfected with control siRNA, SETDB2 siRNA, or SNAI3 siRNA. SETDB2 or SNAI3 depletion led to an increase of metastasis-related markers (**Figures 8G, H**). In addition, the stem cell markers (SOX2, OCT4, and NANOG) were upregulated with the knockdown of SETDB2 or SNAI3 (**Figure 8I**). These results show that SETDB2 and SNAI3 inhibit proliferation, migration, invasive, metastasis, and cell stemness of lung cancer cells.

## 4 Discussion

Lung cancer is one of the most common cancers all over the world and has become a major threat to human health due to its high morbidity and mortality (30). In 2001, the 3-year survival rate of patients with lung cancer was 19%, increasing to 31% in 2015–2017. This is due to the progress in the diagnosis and treatment of NSCLC, including more accurate staging and advanced surgical concepts and techniques (34–37). Therefore, identifying gene markers related to prognosis and exploring potential NSCLC targets are crucial efforts in its diagnosis and treatment, and the discovery of more sensitive and specific biomarkers will improve the diagnosis and treatment of NSCLC.

In the present study, we screened 725 differentially expressed TFs in normal lung tissue and NSCLC. In the WGCNA module analysis of these disease-related TFs, three similar modules were found to be associated with survival state and survival time, from which 118 survival phenotype-related TFs were obtained. Nine survival-related TFs were identified using Cox univariate analysis; five survival-related TFs (SETDB2, SNAI3, SCML4, ZNF540, and ETV1) and the corresponding regression factors were obtained using LASSO regression analysis.

Accumulating evidence showed that TFs play important roles in tumorigenesis, metastasis, and tumor immunity (38–40). SETDB2 promoted methylation (transcriptional repression) of histone H3K9, and SETDB2 was involved in innate immune inflammation and response. *SETDB2* expression was upregulated in M1 macrophages, which killed tumor cells, but not in M2 macrophages, which promoted tumor growth, invasion, and metastasis (41, 42). In addition, SETDB2 was also a glucocorticoid-induced putative epigenetic modifier that promotes the enrichment of glucocorticoid receptor chromatin, and glucocorticoids were found to inhibit the growth of lymphoma (43, 44). SNAI3 is a member of the Snail family. *SNAI3* expression was downregulated in multiple tumors compared to normal tissues. Whereas *SNAI3* expression was found to be associated with good prognosis in breast cancer. SNAI3 had a potential opposite role for SNAI1 and SNAI2 in tumorigenesis and progression (45). *SCML4* expression was shown to correlate with poor prognosis in breast cancer (46). Studies found that SCML4 potentially regulates the immune response and is involved in vascular remodeling (46, 47). *ZNF540* is a zinc-finger protein located on chromosome 19; it interacts with MVP and inhibits the transcriptional activity of the ERK signaling pathway (48, 49). Some studies showed that CpG methylation changes in the *ZNF540* through renal clear carcinoma tissue analysis associated with tumor aggressiveness and patient prognosis (50, 51). The above study showed that ZNF540 is a potential suppressor gene in tumors. ETV1 has a vital role in developing the cardiac conduction system, muscle development, and cerebellar circuit development (52). In prostate cancer, androgen receptor activation mediates *ETV1* expression, activating Twist1, leading to EMT and tumor metastasis (53). Similarly, ETV1 is activated by HER2/Neu in high-risk female tumors (breast, endometrial, and ovarian cancers), mediating the malignant tumor phenotype (54, 55). Overexpression of *ETV1* in various tumors mediates cell growth, invasion, and migration in various tumor cells, leading to tumor progression, metastasis, and drug resistance (52, 55). Therefore, SETDB2, SNAI3, SCML4, and ZNF540 are potential suppressors in tumors, and their specific mechanisms should be further explored.

The immune infiltration of various immune cells was different in the high- and low-score groups, including various regulatory T cells, activated NK cells, monocytes, M0 macrophages, and M1 macrophages, which were important in the TME. Regulatory T cells were immunosuppressive T cells categorized by the expression of *FOXP3*, which hinders effective antitumor immune responses (56, 57). Furthermore, the gut microbiota regulates intertumoral infiltration and NK cell activity to promote pancreatic ductal adenocarcinoma progression. In addition, Tumor-associated macrophages (TAMs) regulate tumor progression, promoting lung cancer cells to assume an M2 (TAM-like) phenotype and, subsequently, EMT and invasion of lung cancer cells (58). Therefore, this predictive model showed that the two groups of tumors had different immune cell profiles and infiltration. This ongoing research intended to predict the effects of immunotherapy and develop new treatment strategies.

In conclusion, we performed WGCNA analysis on NSCLC-related TF genes and mined module TF genes related to survival prognosis, including Cox single factor analysis, to obtain candidate prognostic risk TF genes. This 5-TF prediction model was validated using an additional UCSC dataset. Meanwhile, the cohort was divided into high- and low- score groups according to the model, and the differences in the proportional distribution of immune cells between the high- and low- score groups were compared. In addition, the roles of SETDB2, SNAI3, SCML4, ZNF540, and ETV1 were validated in multiple datasets and human lung cancer cell lines. The method provided in this study was beneficial for screening patients with NSCLC with poor prognoses to achieve early detection, early treatment, and improved survival. This study had several limitations, more samples were needed for further verification and optimization in the future.

## Data availability statement

The datasets presented in this study can be found in online repositories. The names of the repository/repositories and accession number(s) can be found in the article/**Supplementary Material**.

## Author contributions

WH and YanW contributed the study design. JYZ were involved in the acquisition of data, prepared all figures and tables, and wrote the manuscript. JYZ, YNW, YY, JZ, MZ performed the experiments. BY, HQ, YongW, HY, XT analyzed the data. JYZ, WH, and YanW wrote the original draft. All authors approved this work for publication. All authors contributed to the article.
